# The Auxiliary Role of the Amidase Domain in Cell Wall Binding and Exolytic Activity of Staphylococcal Phage Endolysins

**DOI:** 10.3390/v10060284

**Published:** 2018-05-25

**Authors:** Bokyung Son, Minsuk Kong, Sangryeol Ryu

**Affiliations:** 1 Laboratory of Molecular Food Microbiology, Department of Food and Animal Biotechnology, Seoul National University, Seoul 08826, Korea; sonbk0722@gmail.com (B.S.); kongmin1@snu.ac.kr (M.K.); 2 Department of Agricultural Biotechnology and Center for Agricultural Biomaterials, Seoul National University, Seoul 08826, Korea

**Keywords:** *Staphylococcus aureus*, endolysin, amidase domain, cell wall binding

## Abstract

In response to increasing concern over antibiotic-resistant *Staphylococcus aureus*, the development of novel antimicrobials has been called for, with bacteriophage endolysins having received considerable attention as alternatives to antibiotics. Most staphylococcal phage endolysins have a modular structure consisting of an N-terminal cysteine, histidine-dependent amidohydrolases/peptidase domain (CHAP), a central amidase domain, and a C-terminal cell wall binding domain (CBD). Despite extensive studies using truncated staphylococcal endolysins, the precise function of the amidase domain has not been determined. Here, a functional analysis of each domain of two *S. aureus* phage endolysins (LysSA12 and LysSA97) revealed that the CHAP domain conferred the main catalytic activity, while the central amidase domain showed no enzymatic activity in degrading the intact *S. aureus* cell wall. However, the amidase-lacking endolysins had reduced hydrolytic activity compared to the full-length endolysins. Comparison of the binding affinities of fusion proteins consisting of the green fluorescent protein (GFP) with CBD and GFP with the amidase domain and CBD revealed that the major function of the amidase domain was to enhance the binding affinity of CBD, resulting in higher lytic activity of endolysin. These results suggest an auxiliary binding role of the amidase domain of staphylococcal endolysins, which can be useful information for designing effective antimicrobial and diagnostic agents against *S. aureus*.

## 1. Introduction

*Staphylococcus aureus* is a Gram-positive facultative anaerobe that frequently colonizes the nose, skin, or gastrointestinal tract [1]. *S. aureus* has long been recognized as an important pathogen causing a wide range of infections, from superficial skin infections to life threatening diseases such as bacteremia, necrotizing pneumonia, and endocarditis [2]. Although these infections were historically treatable with standard antibiotics, the emergence and spread of multidrug-resistant forms of *S. aureus* have greatly limited treatment options. Therefore, new approaches to controlling *S. aureus* are urgently required [1,3,4]. Bacteriophages produce highly evolved lytic enzymes, called endolysins, which disrupt host bacterial cell walls to release their progeny [5]. Due to the strong lytic activity and high specificity, use of endolysins has been proposed as an alternative antimicrobial for treatment of staphylococcal infections [6]. Endolysins of Gram-positive bacteria generally have a modular structure consisting of a catalytic domain (s) and a cell wall binding domain (CBD), while about 75% of the endolysins of staphylococcal phages have three distinct domains: an N-terminal cysteine-, histidine-dependent amidohydrolase/peptidase (CHAP) domain, a central *N*-acetylmuramoyl-l-alanine amidase (Ami_2 or Ami_3) domain, and a C-terminal SH3b domain as a CBD [7]. Several truncation studies of *S. aureus* endolysins have reported that the CHAP domains confer most of the lytic activity of the endolysin by cleaving the multiple peptide bonds of peptidoglycan [8,9,10]. On the other hand, the central amidase domains of *S. aureus* endolysins showed little or no lytic activity by themselves [8,9,11,12]. Although it is common for one catalytic domain of endolysins to have dominant activity and the silent_domain can act in a cooperative manner to cleave multiple peptidoglycan bonds [8,11,13], the exact role of the amidase domains of *S. aureus* endolysins is still unclear.

Here, we elucidated the role of the amidase domains of two *S. aureus* phage endolysins (LysSA12 and LysSA97). The amidase domains of both endolysins help CBDs bind to host cells more efficiently and thereby enhance the overall lytic activity of the endolysin. This is, to the best of our knowledge, the first report elucidating the auxiliary role of amidase in cell wall binding.

## 2. Materials and Methods

### 2.1. Bacterial Strains and Growth Conditions

All tested *S. aureus* cells were grown in tryptic soy broth (TSB) medium (Difco, Detroit, MI, USA) at 37 °C with shaking. *Escherichia coli* DH5α and BL21 (DE3) star were grown in LB broth (Difco, Detroit, MI, USA) at 37 °C and used in the cloning and expression of proteins, respectively.

### 2.2. In Silico Analysis of Staphylococcal Endolysins

The sequences of LysSA12 and LysSA97 were analyzed. Their domain composition was investigated using the BLAST (http://blast.ncbi.nlm.nih.gov/Blast.cgi), Interproscan 5 (http://www.ebi.ac.uk/interpro), and Pfam 28.0 programs (http://pfam.xfam.org) [14,15,16]. Amino acid sequence alignments of the endolysins were performed using ClustalX2.1 [17]. The molecular weight and isoelectric point of the proteins were calculated using the Compute pI/Mw program (http://www.expasy.ch/tools/pi_tool.html) [18].

### 2.3. Cloning, Expression, and Purification of S. aureus Endolysin Derivatives and EGFP Fusion Proteins

To clone the endolysins and their derivatives, genes were amplified from the genome of the bacteriophages SA12 (GenBank accession No. KC677663) and SA97 (GenBank accession No. KJ716334) using the oligonucleotides listed in Table 1 [7,19,20]. The PCR product was cloned into pET28a (Novagen, Madison, WI, USA) carrying an *N*-terminal His-tag sequence. All plasmids used in this study are listed in Table 1. The correctly cloned plasmid was transformed into competent *E. coli* BL21 (DE3). Expression of the recombinant proteins was induced with 0.5 mM IPTG (isopropyl-β-d-thiogalactopyranoside) at OD_600_ (optical density at 600 nm) = 0.7, followed by incubation for additional 20 h at 18 °C. Bacterial cells were suspended in lysis buffer (50 mM sodium phosphate, 300 mM sodium chloride and 30% glycerol; pH 8.0) and disrupted by sonication at a duty cycle of 25% and output control of 5 (Sonifier 250, Branson, Danbury, CT, USA). After centrifugation (20,000× *g*, 30 min), the supernatant was passed through Ni-NTA agarose (Qiagen, Hilden, Germany), and purification of the recombinant proteins was performed according to the manufacturer’s instructions. After the buffer was changed to storage buffer (50 mM sodium phosphate, 300 mM NaCl, and 30% glycerol; pH 8.0) using PD Miditrap G-25 (GE Healthcare, Chicago, IL, USA), the purified protein was stored at −80 °C until use. To generate EGFP fusion proteins, the endolysin derivatives were cloned into EGFP-harboring pET28a [21]. Each protein was expressed and purified as described above.

### 2.4. Lytic Activity Assay of Endolysin and Their Derivatives

The lysis activity of endolysins and their derivatives was assessed using a turbidity reduction assay [22]. Bacterial cells grown to the exponential phase were re-suspended in reaction buffer (50 mM sodium phosphate, 300 mM NaCl; pH 8.0). Purified endolysins and their derivatives were then added to the cell suspension at a final concentration of 0.3 μM, and the OD reduction of cells was measured over time using a SpectraMax i3 multimode microplate reader (Molecular Devices, Sunnyvale, CA, USA). The assay on peptidoglycan was performed as described above. Purified peptidoglycan was prepared by a method described by Kuroda et al. [23].

### 2.5. EGFP Fusion Protein Binding Assay

The binding of each EGFP fusion protein to *S. aureus* cells was measured as previously described [24]. Bacterial cells, cultivated to an early exponential phase, were harvested and re-suspended in Dulbecco’s phosphate-buffered saline (PBS). The cells were incubated with EGFP fusion protein for 5 min at room temperature. The mixture was washed twice with PBS to remove unbound protein and was transferred to a 96-well plate to measure fluorescence using a SpectraMax i3 multimode microplate reader with excitation at 485 nm and emission at 535 nm. The OD_600_ of cells was measured, and fluorescence was normalized by calculating the whole-cell fluorescence per OD_600_. Relative binding capacity was derived by comparing to the highest measured value and is presented as relative fluorescence intensity (RFI). In addition, target bacteria labelled with EGFP fusion protein were examined by fluorescence microscopy (DE/Axio Imager A1 microscope, Carl Zeiss, Oberkochen, Germany) using a GFP filter set (470/40 nm excitation, 495 nm dichroic, 525/50 nm emission).

## 3. Results

### 3.1. Modular Structure of LysSA12

**Figure 1:**
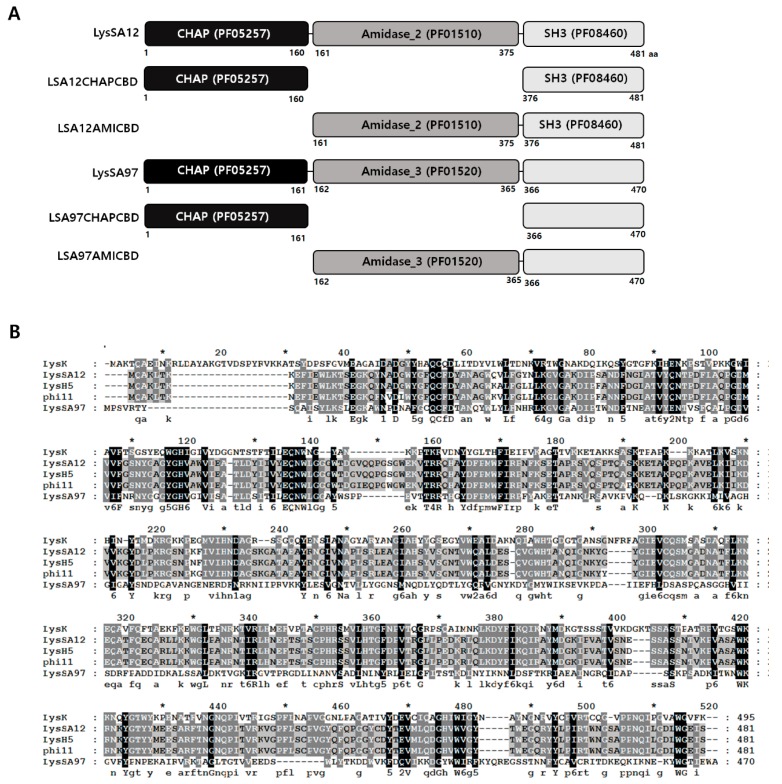
Modular structure of LysSA12 and LysSA97. (**A**) Schematic representations of LysSA12, LysSA97, and their deletion constructs. The numbers indicate the initial and final amino acids in the domains as determined using the Pfam domain database. CHAP domain, black box; amidase domain, dark grey box; CBD, light grey box; (**B**) Sequence alignment of LysSA12 and LysSA97 with other related endolysins. LysK, *S. aureus* phage K endolysin; LysH5, *S. aureus* phage vB_SauS-phiIPLA88 endolysin; phi11, *S. aureus* phage phi11 endolysin. Conserved and identical residues are shaded in gray (dark gray, >70% conserved; light gray, >40% conserved) and black, respectively. The conserved Cys-His-Asn triad is indicated by triangles.

Bioinformatics analysis revealed that the endolysin of phage SA12, LysSA12 (Genbank accession No. AGO49867.1), consists of an N-terminal CHAP (PF05257) domain (LSA12CHAP), a central amidase_2 (PF01510) domain (LSA12AMI), and a C-terminal CBD (LSA12CBD) with homology to the SH3 domain (PF08460) [19,25]. LysSA12 showed an overall 98% amino acid identity with endolysin LysH5, an endolysin (Genbank accession No. EU 573240.1) from *S. aureus* phage phi-SauS-IPLA88. [26], and a 96% identity with endolysin phi11 (Genbank accession No. NC_004615.1) found in genomic DNA of *S. aureus* NCTC8325 [11]. LysK (Genbank accession No. AAO47477.2), an endolysin from staphylococcal phage K, showed only limited amino acid sequence similarity (37% identity) to LysSA12 [27]. LysSA12 has a conserved Cys-His-Asn catalytic triad in its CHAP domain, and these residues are conserved in LysH5, phi11, and LysK (Figure 1B).

### 3.2. Expression and Purification of LysSA12 Derivatives

**Figure 2:**
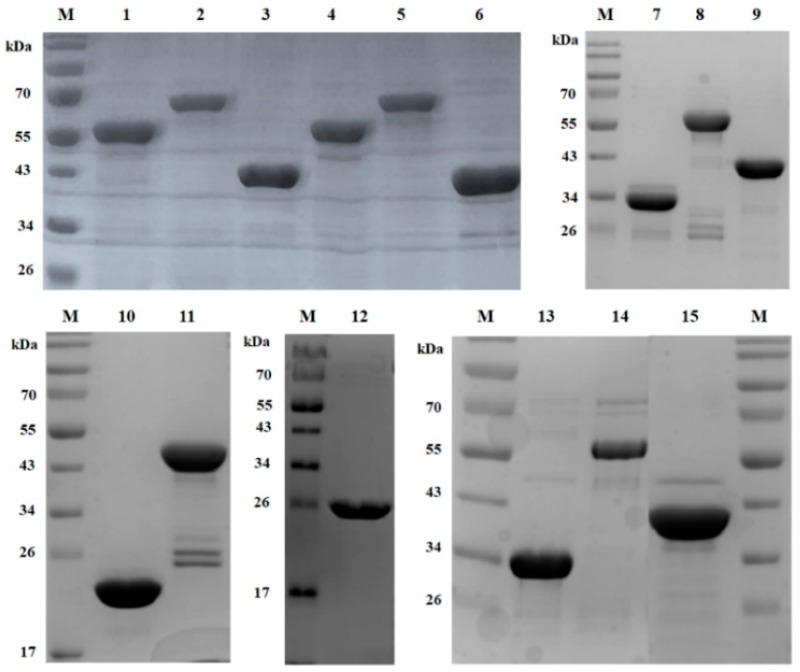
SDS-polyacrylamide gel electrophoresis analysis of LysSA12, LysSA97, and their derived proteins. Proteins were purified by nickel-nitrilotriacetic acid affinity chromatography and analyzed using 12% SDS-polyacrylamide gel electrophoresis. Lane M, protein ladder; lane 1, EGFP_LSA12AMI; lane 2, EGFP_LSA12AMICBD; lane 3, EGFP_LSA12CBD; lane 4, EGFP_LSA97AMI; lane 5, EGFP_LSA97AMICBD; lane 6, EGFP_LSA97CBD; lane 7, LSA12 CHAPCBD; lane 8, LysSA12; lane 9, LSA12AMICBD; lane 10, LSA12CHAP; lane 11, LSA12CHAPAMI; lane 12, LSA12AMI; lane 13, LSA97CHAPCBD; lane 14 LysSA97; lane 15, LSA97AMICBD.

Since a defined function for the amidase domain in endolysins containing both CHAP and amidase domains is not known, the influence of the amidase domain on enzymatic activity or on binding ability of endolysin was studied by comparing the lytic activities and binding abilities of various combinations of the three domains of LysSA12. The truncated proteins consisting of LSA12CHAP, LSA12AMI, LSA12CHAP plus LSA12AMI (LSA12CHAPAMI), LSA12CHAP plus LSA12CBD (LSA12CHAPCBD), and LSA12AMI plus LSA12CBD (LSA12AMICBD) were constructed based on domain composition (Figure 1B), and their enzymatic activities were compared with those of intact LysSA12. In addition, three EGFP fusion proteins containing LSA12AMI, LSA12AMICBD, and LSA12CBD, were constructed to test their binding ability. The proteins were overexpressed in *E. coli* using the pET-28a vector, and the purified proteins migrated as a single band of the expected molecular mass on SDS-PAGE (Figure 2).

### 3.3. Lytic Activities of LysSA12 and Its Truncated Proteins

**Figure 3:**
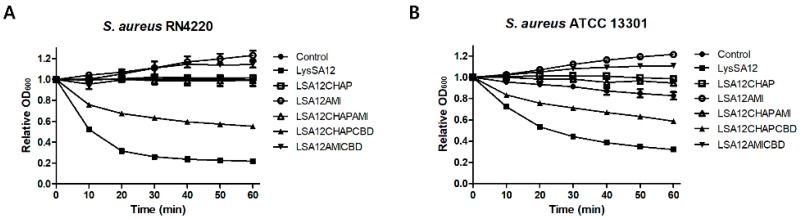
Activity comparison of LysSA12 and its derivate domains against *S. aureus* RN4220 and *S. aureus* 13301. Equimolar concentrations (0.3 μM) of the purified enzymes expressed from the full length and truncated proteins were added to a 1 mL suspension of (**A**) *S. aureus* RN4220 and (**B**) *S. aureus* ATCC 13301.

Turbidity reduction assays with LysSA12 and its truncated proteins were conducted against the exponentially growing *S. aureus* cells. LSA12AMICBD and LSA12AMI did not inhibit the growth of cells, but rather caused a slight increase in the OD values (Figure 3A,B). It has been reported that the negatively charged teichoic acid and lipoteichoic acid on the surface of *S. aureus* cells can interact with positively charged proteins to increase the OD value [28]. The calculated pI values of the truncated proteins of LysSA12 with no lytic activity are 5.3 for LSA12CHAP, 8.73 for LSA12CHAPAMI, 9.18 for LSA12AMICBD, and 9.38 for LSA12AMI, suggesting that the positively charged LSA12AMICBD and LSA12AMI with high pI values caused an increase in OD value as reported by Takano et al. [28]. LSA12CHAP, LSA12AMI, and LSA12CHAPAMI barely lysed the intact *S. aureus* cells, whereas LSA12CHAPCBD and the full length LysSA12 resulted in clear lysis, suggesting that the both CHAP and a C-terminal CBD are necessary to exert the lytic activity of LysSA12. LSA12CHAPCBD showed 2-fold reduced activity compared with wild-type LysSA12 at equimolar concentrations. These findings demonstrate that the highest exolytic activity of LysSA12 requires the amidase domain.

### 3.4. Amidase Domain Helps CBD Bind to Intact Cells

**Figure 4:**
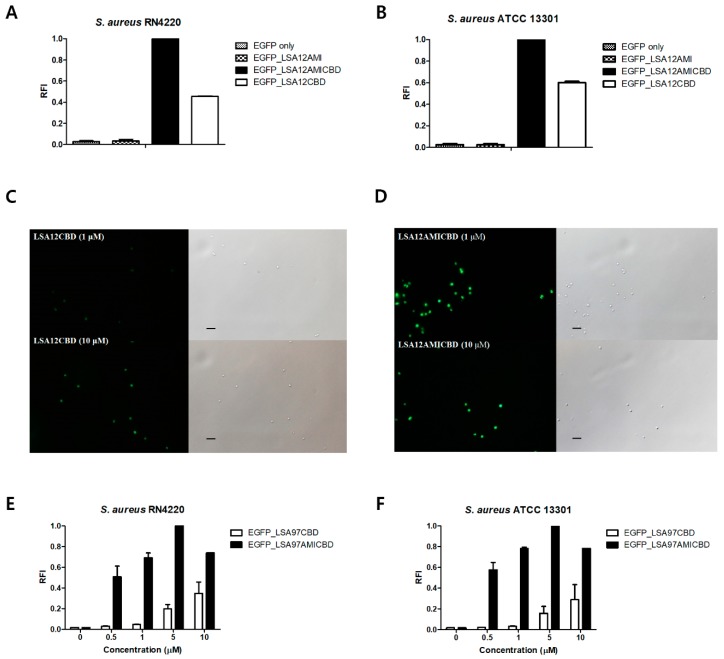
Binding activity comparison among EGFP-fused LysSA12 derivatives. Relative cell binding activities of 1 μM of EGFP_LSA12AMI, EGFP_LSA12AMICBD, and EGFP_LSA12CBD toward (**A**) *S. aureus* RN4220 and (**B**) *S. aureus* ATCC 13301. Optical and florescent images of *S. aureus* RN4220 after the addition of (**C**) EGFP_LSA12CBD and (**D**) EGFP_LSA12AMICBD at 1 μM (top) and 10 μM (bottom). Relative cell binding activities of EGFP_LSA12CBD and EGFP_LSA12AMICBD were measured with (**E**) *S. aureus* RN4220 and (**F**) *S. aureus* ATCC 13301 at different concentrations. EGFP did not show binding to *S. aureus*. The data shown are the mean values from three independent measurements and the error bars represent the standard deviations. The scale bar represents 2 μm.

To elucidate the role of the amidase domain in exolysis of target bacteria, the binding activities of EGFP_LSA12AMI, EGFP_LSA12CBD, and EGFP_LSA12AMICBD were examined by measuring the RFI of *S. aureus* after mixing bacterial cells with the EGFP fusion proteins (Figure 4). Among the EGFP fusion proteins tested, EGFP_LSA12AMICBD showed the highest ability to bind to *S. aureus*, while EGFP_LSA12AMI had negligible binding to the *S. aureus* cells. EGFP_LSA12AMICBD exhibited approximately 2-fold enhanced binding capacity to *S. aureus* cells relative to EGFP_LSA12CBD (Figure 4A,B). These results were confirmed by fluorescent microscopy images, which clearly showed that EGFP_LSA12AMICBD displayed greater binding to the *S. aureus* cells relative to EGFP_LSA12CBD (Figure 4C,D). We also compared the binding activities of LSA12AMICBD and LSA12CBD at various concentrations (0.5, 1, 5, and 10 μM). Compared to EGFP_LSA12CBD, EGFP_LSA12AMICBD showed more than 2-fold stronger binding activity at all concentrations tested, implying that the amidase domain increases the binding activity of CBD (Figure 4E,F). Taken together, these findings indicate that the amidase domain of LysSA12 increases the lytic activity of the CHAP domain by enhancing accessibility of the CHAP domain to the target bacteria.

### 3.5. Role of the LysSA97 Amidase Domain

**Figure 5:**
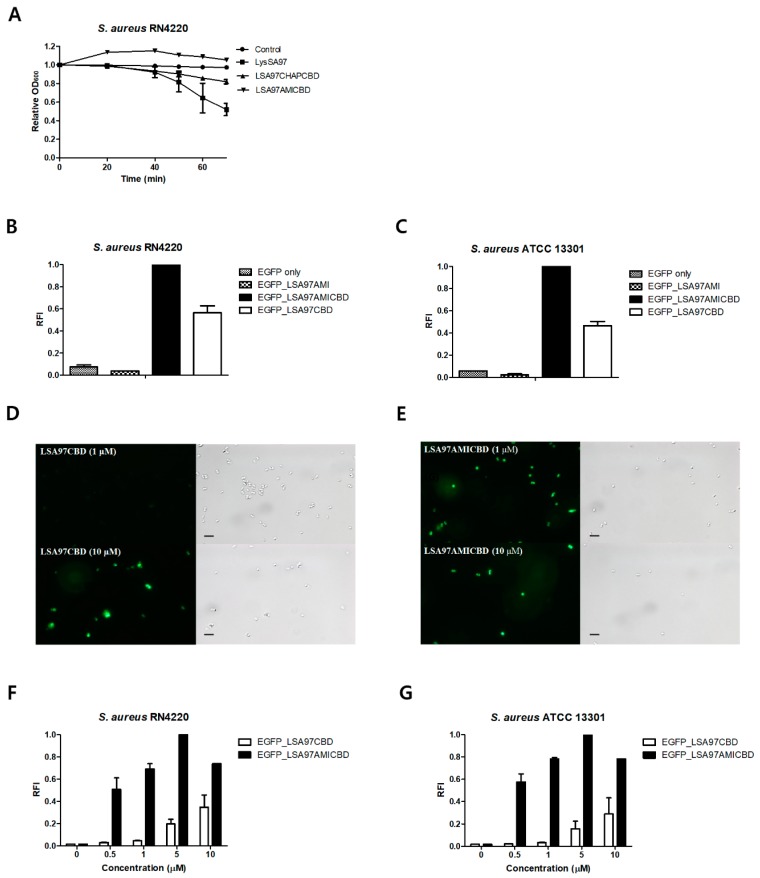
Determination of the role of the LysSA97 amidase domain. (**A**) Activity comparison of LysSA97 and LSA97CHAPCBD against *S. aureus* RN4220. Equimolar concentrations (2.0 μM) of purified enzymes from the full length and truncation constructs were added to a 1 ml suspension of intact *S. aureus* RN4220, cells and the relative decrease in turbidity was monitored. Relative cell binding activities of 10 μM of EGFP_LSA97AMI, EGFP_LSA97AMICBD, and EGFP_LSA97CBD with (**B**) *S. aureus* RN4220 and (**C**) *S. aureus* ATCC 13301. Optical and florescence images of *S. aureus* RN4220 after the addition of (**D**) EGFP_LSA97CBD and (**E**) EGFP_LSA97AMICBD at 1 μM (top) and 10 μM (bottom). At different concentrations, the relative cell binding activities of EGFP_LSA97CBD and EGFP_LSA97AMICBD were measured with (**F**) *S. aureus* RN4220 and (**G**) *S. aureus* ATCC 13301. The data shown are the mean values from three independent measurements and the error bars represent the standard deviations. The scale bar represents 2 μm.

To further investigate the role of the amidase domain in other *S. aureus* endolysins, the LysSA97 endolysin (Genbank accession No. AHZ95694.1) isolated from staphylococcal phage SA97 was studied (Figure 5) [20]. Bioinformatics analysis predicted that LysSA97 would be an *N*-acetylmuramoyl-l-alanine amidase composed of CHAP, amidase_3, and a novel CBD not homologous to any of the reported CBDs. These findings indicate that LysSA12 and LysSA97 belong to different staphylococcal endolysin groups based on their domain compositions (Figure 1A). As in the analysis of LysSA12 and its truncated proteins, LSA97AMICBD did not have any lytic activity against intact *S. aureus* cells, and LSA97CHAPCBD showed reduced lytic activity compared to the full-length LysSA97 (Figure 5A). The binding assay revealed that EGFP_LSA97AMICBD exhibited the highest cell binding activity among the constructs tested (Figure 5B,C), which was approximately 15-fold higher than LSA97CBD at 1.0 μM (Figure 5D–G). These results suggest that the amidase domain of LysSA97 also improves the weak binding of CBD and thereby enhances the lytic activity of the endolysin.

## 4. Discussion

Improvement of endolysin’s antibacterial activity by the increased binding ability of CBD has been reported in several previous studies [29,30,31]. In this study, the role of the amidase domain of staphylococcal endolysins was investigated and was found to help CBD bind intact bacterial cells, thereby contributing to the overall lytic activity of the endolysin. Most endolysins from staphylococcal phages and some streptococcal phage endolysins are composed of three domains: a CHAP domain, an amidase domain, and a CBD [6,32]. The CHAP domain is known to be responsible for the major catalytic activity of the endolysin in degrading cell wall peptidoglycan, exhibiting both amidase and endopeptidase activity [8,33]. Sometimes, there is an inactive domain among two different EADs in the same endolysin [12,34]. For example, the isolated amidase domain of Φ11 endolysin [11], the glycosidase domains from the λSa2 prophage endolysin [30] or B30 endolysin [35], and the amidase domain in LysK [8] are unable to hydrolyze bacterial cells. Consistent with these previous data, LSA12CHAPCBD and LSA97CHAPCBD displayed lytic activity against the *S. aureus* cells, while LSA12AMICBD and LSA97AMICBD did not. These findings raised questions about the function of the central amidase domain of endolysins.

Interestingly, the removal of the amidase domains from both LysSA12 and LysSA97 reduced their lytic activity, suggesting that the amidase domains are required for full lytic activity. Similar results have been reported for LysK; amidase deletion reduced the activity of LysK as measured in the turbidity reduction assay using equimolar amount of proteins [8]. To specify the role of the amidase domain in endolysins containing both CHAP and amidase domains, its effect on the binding activity of endolysin was evaluated using EGFP fusion proteins. Surprisingly, this binding assessment showed that EGFP_LSA12AMICBD had stronger binding ability against staphylococcal cells than EGFP_LSA12CBD, suggesting that the amidase domain of LysSA12 might increase the binding affinity of LSA12CBD. This unexpected result was also observed in LysSA97, which has a different domain composition than LysSA12. The enhancing effect of the amidase domain on cell wall binding was more evident in LysSA97 endolysin due to the relatively low binding capacity of LSA97CBD. Based on these results, we suggest the auxiliary role of the amidase domain in cell wall binding and exolytic activity of staphylococcal phage endolysins even though the results obtained with the two endolysins in this study might not be extended to all staphylococcal endolysins.

Several studies have shown that the central amidase domain of endolysins has little or no activity and have proposed potential roles of the amidase domain. Donovan and Foster-Frey pointed out that the apparent lack of activity of the amidase domain may be due to inherent differences in cleavage between lysis from within and lysis from without [25]. Indeed, LSA12AMICBD showed faint lytic activity on the purified *S. aureus* peptidoglycan (Figure S1) while no activity was observed against the intact cells. This discrepancy, which has also been reported in several other studies [11,36], was possibly due to the absence of secondary cell wall polymers, including teichoic acids and cell wall polysaccharides, and/or wall associated proteins in the purified peptidoglycan [37,38,39]. We propose that the lack of LSA12AMICBD lytic activity against the intact cells might result from steric interference of the secondary cell wall structure. Furthermore, Becker and colleagues speculated that cofactors produced during the phage lytic cycle or peptidoglycan fragments created by the initial CHAP digestion may be required for the amidase domain to exert its optimal activity [8]. Our data demonstrated that two different amidase domains of *S. aureus* phage endolysins facilitate binding of each CBD to host cell walls, leading to maximal lytic activity of these endolysins. Considering that the amidase domain alone did not show any cell binding capacity, it could be that the amidase domains help with and stabilize the folding of the CBD or act as a spacer between the CHAP domain and CBD, enabling the CBD to interact with their cognate target cells efficiently. Further structural and biophysical studies will be needed to fully understand the molecular mechanism of enhanced binding activity provided by the amidase domain.

In summary, several truncated derivatives of LysSA12 and LysSA97 were generated to identify the function of each domain. The amidase domains of both staphylococcal endolysins enhanced the overall exolytic activity of enzymes by helping CBD bind its target cells more efficiently. These molecular studies will be helpful in the creation of novel chimeric proteins for preventing and treating *S. aureus* infections. Furthermore, the newly constructed fusion proteins containing the amidase domain and CBD that have higher binding ability to *S. aureus* can be used as promising materials for the development of diagnostic agents.

## Figures and Tables

**Table 1:** Plasmids and primers used in this study.

**Plasmids**		
	**Description**	**Reference**
pET28a	Kan^r^, T7 promoter, His-tagged expression vector	Novagen, Wisconsin, SA
pET28a-EGFP	pET28a with EGFP	This study
pET28a-LysSA12	pET28a with LysSA12 (56 kDa)	[19]
pET28a-LSA12CBD	pET28a with LSA12CBD	This study
pET28a-LSA12CHAP	pET28a with LSA12CHAP (20 kDa)	This study
pET28a-LSA12CHAPCBD	pET28a-LSA12CBD with LSA12CHAP (32 kDa)	This study
pET28a-LSA12AMICBD	pET28a with LSA12AMICBD (44 kDa)	This study
pET28a-LysSA97	pET28a with LysSA97 (56 kDa)	[19]
pET28a-LSA97CBD	pET28a with LSA97CBD	This study
pET28a-LSA97CHAPCBD	pET28a-LSA97CBD with LSA97CHAP (33 kDa)	This study
pET28a-LSA97AMICBD	pET28a with LSA97AMICBD (38 kDa)	This study
pET28a-EGFP_LSA12CBD	pET28a-EGFP with LSA12CBD (42 kDa)	This study
pET28a-EGFP_LSA12AMICBD	pET28a-EGFP with LSA12AMICBD (66 kDa)	This study
pET28a-EGFP_LSA12AMI	pET28a-EGFP with LSA12AMI (54 kDa)	This study
pET28a-EGFP_LSA97CBD	pET28a-EGFP with LSA97CBD (42 kDa)	[7]
pET28a-EGFP_LSA97AMICBD	pET28a-EGFP with LSA97AMICBD (65 kDa)	This study
pET28a-EGFP_LSA97AMI	pET28a-EGFP with LSA97AMI (52 kDa)	This study
**Primers (5′→3′) ^a^**		
	**Sequence**	**Purpose**
BamH1_LSA12CHAP_F	AAA GGA TCC ATGC AAG CAA AAC TAA CTA AAA A	pET28a-LSA12CHAP and pET28a-LSA12CHAPCBD construction
LSA12CHAP_Sal1_R	TTT GTC GAC TGA TCG TGG AGC TGT TTC GCT T	pET28a-LSA12CHAP construction
LSA12CHAP_EcoR1_R	TTT GAA TTC TGA TCG TGG AGC TGT TTC GCT T	pET28a-LSA12CHAPCBD construction
BamH1_LSA12AMI_F	AAA GGA TCC GTA CAA TCT CCT ACG CAA GCA	pET28a-LSA12AMI, pET28a-LSA12AMICBD, pET28a-EGFP_LSA12AMI and pET28a-EGFP_LSA12AMICBD construction
LSA12AMI_Sal1_R	TTT GTC GAC ACT TGA AGC GCT TGA CTC ATT AG	pET28a-LSA12AMI and pET28a-EGFP_LSA12AMI construction
EcoR1_LSA12CBD_F	AAA GAA TTC TCA AGT AAT ACA GTT AAA CCA GT	pET28a-LSA12CBD construction
LSA12CBD_Sal1_R	TTT GTC GAC ACT GAT TTC TCC CCA TAA GT	pET28a-LSA12CBD, pET28a-LSA12AMICBD and pET28a-EGFP_LSA12AMICBD construction
BamH1_LSA97CHAP_F	AAA GGA TCC ATG CCG TCG GTT AGG ACA TAC AG	pET28a-LSA97CHAP and pET28a-LSA97CHAPCBD construction
LSA97CHAP_Sal1_R	TTT GTC GAC TTC TTT TGC GTA GAA TGG ACG GAT	pET28a-LSA97CHAPconstruction
LSA97CHAP_EcoR1_R	TTT GAA TTC TTC TTT TGC GTA GAA TGG ACG GAT	pET28a-LSA97CHAPCBD construction
BamH1_LSA97AMI_F	AAA GGA TCC CAA GAT AAG TTA TCA AAA GGT AAA	pET28a-LSA97AMICBD, pET28a-EGFP_LSA97AMICBD and pET28a-EGFP_LSA97AMI construction
LSA97AMI_Sal1_R	TTT GTC GAC ACT ACT TGG CGC ATC AAT TTG TC	pET28a-EGFP_LSA97AMI construction
EcoR1_LSA97CBD_F	AAA GAA TTC AGT AGT AAG CCA AGC GCT GAC AA	pET28a-LSA97CBD construction
LSA97CBD_Sal1_R	TTT GTC GAC TTA AGC CCA CTC AAT CGT GCC CCA	pET28a-LSA97CBD, pET28a-LSA97AMICBD and pET28a-EGFP_LSA97AMICBD construction
Nde1_EGFP_F	AAA CAT ATG ATG GTG AGC AAG GGC GAG GA	pET28a-EGFP construction
EGFP_BamH1_R	TTT GGA TCC CTT GTA CAG CTC GTC CAT GCC G	

^a^ Restriction sites are underlined.

## Supplementary Materials

The following are available online at http://www.mdpi.com/1999-4915/10/6/284/s1, Figure S1: Activity of the LysSA12 and LSA12AMICBD against purified peptidoglycan of *S. aureus* RN4220 and *S. aureus* ATCC 13301.
