# Supplementary material for: The Auxiliary Role of the Amidase Domain in Cell Wall Binding and Exolytic Activity of Staphylococcal Phage Endolysins
Source: Viruses. 2018 May 25;10(6):284. doi: 10.3390/v10060284 (PMC6024855; doi:10.3390/v10060284)
Supplement: Supplementary file 1 [file viruses-10-00284-s001.pdf]

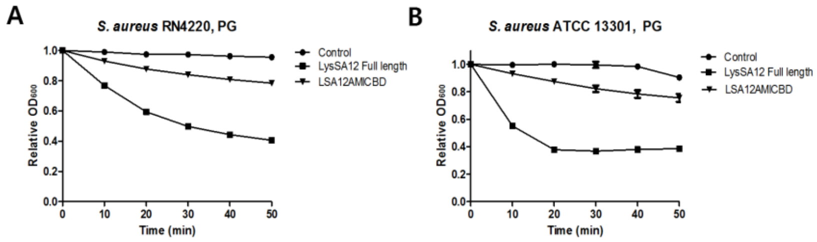

Figure S1. Activity of the LysSA12 and LSA12AMICBD against purified peptidoglycan of *S. aureus* RN4220 and *S. aureus* ATCC 13301. Equimolar concentrations (0.3 μM) of the purified enzymes were added to a 1 ml purified peptidoglycan of (A) *S. aureus* RN4220 and (B) *S. aureus* ATCC 13301.
